# Smoking Status and Functional Outcomes in Young Stroke

**DOI:** 10.3389/fneur.2021.658582

**Published:** 2021-09-01

**Authors:** Cheng-Loong Liang, Han-Jung Chen, Yi-Che Lee, Cheng-Chun Wu, Chon-Haw Tsai, Po-Lin Chen, Wei-Lun Chang, Po-Yen Yeh, Cheng-Yu Wei, Ming-Jun Tsai, Yu Sun, Chih-Hao Lin, Jiunn-Tay Lee, Ta-Chang Lai, Li-Ming Lien, Mei-Chen Lin, Cheng-Li Lin, Hao-Kuang Wang, Chung Y. Hsu

**Affiliations:** ^1^School of Medicine, I-Shou University, Kaohsiung, Taiwan; ^2^Department of Neurosurgery, E-Da Hospital, Kaohsiung, Taiwan; ^3^Department of Nephrology, E-Da Hospital, Kaohsiung, Taiwan; ^4^Division of Neurology, China Medical University Hospital, Taichung, Taiwan; ^5^Neurological Institute, Taichung Veterans General Hospital, Taichung, Taiwan; ^6^Department of Neurology, Show Chwan Memorial Hospital, Changhua, Taiwan; ^7^Department of Neurology, St. Martin de Porres Hospital, Chiayi, Taiwan; ^8^Department of Neurology, Chang Bing Show Chwan Memorial Hospital, Changhua, Taiwan; ^9^Department of Neurology, Tainan Municipal An-Nan Hospital-China Medical University, Tainan, Taiwan; ^10^Department of Neurology, En Chu Kong Hospital, New Taipei, Taiwan; ^11^Department of Neurology, Lin Shin Hospital, Taichung, Taiwan; ^12^Department of Neurology, Tri-Service General Hospital, Taipei, Taiwan; ^13^Department of Neurology, Cheng Hsin General Hospital, Taipei, Taiwan; ^14^Department of Neurology, Shin Kong Wu Ho Su Memorial Hospital, Taipei, Taiwan; ^15^Management Office for Health Data, China Medical University Hospital, Taichung, Taiwan; ^16^College of Medicine, China Medical University, Taichung, Taiwan; ^17^Department of Neurology, China Medical University Hospital, Taichung, Taiwan; ^18^Graduate Institute of Clinical Medical Science, China Medical University, Taichung, Taiwan

**Keywords:** stroke, smoking, Taiwan Stroke Registry, smoking cessation, proportional hazards regression analysis

## Abstract

**Objective:** Stroke in young adults is uncommon, and the etiologies and risk factors of stroke in young adults differ from those in older populations. Smoker's paradox is an unexpected favorable outcome, and age difference is used to explain the association between smoking and the favorable functional outcome. This study aimed to investigate the existence of this phenomenon in young stroke patients.

**Methods:** We analyzed a total of 9,087 young stroke cases registered in the nationwide stroke registry system of Taiwan between 2006 and 2016. Smoking criteria included having a current history of smoking more than one cigarette per day for more than 6 months. After matching for sex and age, a Cox model was used to compare mortality and function outcomes between smokers and non-smokers.

**Results:** Compared with the non-smoker group, smoking was associated with older age, higher comorbidities, and higher alcohol consumption. Patients who report smoking with National Institutes of Health Stroke Scale scores of 11–15 had a worse functional outcome (adjusted odds ratio, 0.81; 95% confidence interval, 0.76 – 0.87).

**Conclusion:** Smokers had a higher risk of unfavorable functional outcomes at 3 months after stroke, and therefore, we continue to strongly advocate the importance of smoking cessation.

## Introduction

Stroke in young adults is relatively uncommon (1–4). Although the prognosis of young stroke is generally considered benign, young adults with stroke are at a higher risk of recurrent stroke and mortality than their healthy peers. Most survivors between 20 and 50 years may have emotional, social, or physical sequelae that impair their quality of life (4, 5). In addition, young stroke victims are often responsible for providing child care or generating income for their families. Therefore, young stroke is a major health and socioeconomic problem.

Smoking is one of the biggest public health threats associated with many chronic diseases, such as cardiovascular disease and cancer (6–8). More importantly, smoking is also aggregated with other adverse behaviors, such as chewing bet-but and drinking alcohol. However, several recent studies observed an improved outcome in smokers with antithrombotic therapy after an index cardiovascular event, a phenomenon called “smoker's paradox” (9–13). Sample sizes in these studies are small (10, 11). Moreover, no study has examined the effects of pre-stroke smoking on post-stroke long-term outcome.

In Taiwan, about 24,000 smokers die of smoking and nearly 2,600 non-smokers die of second-hand smoke each year. Since the enactment of the Tobacco Hazards Prevention Act (THPA) in 1997, smoking prevalence has decreased. However, Taiwan still records a 15% adult smoking rate. The purpose of this study was to evaluate the effect of smoking status on stroke outcome in young stroke.

## Methods

### Standard Protocol Approvals, Registrations, and Patient Consents

The Taiwan Stroke Registry (TSR) program was started in 2006. Until now, 56 hospitals participated in this registration project. This study obtained ethical approval from China Medical University (CMUH104-REC2-115) and the institutional review boards of the collaborating hospitals. The TSR enrolls patients who were taken to hospital due to stroke, including ischemic stroke, intracerebral hemorrhage, subarachnoid hemorrhage, and transient ischemic attack (14–16). The National Institutes of Health Stroke Scale (NIHSS), and the modified Rankin Scale (mRS) were used to check the severity and outcome by trained neurologists in hospital. After discharge, study nurses followed up those patients every 3 months for at least 12 months. The Taiwan Stroke Registry investigators are listed in Appendix 1.

### Study Design and Eligibility Criteria

All registered patients aged between 20 and 50 years from August 1, 2006, to May 20, 2016, were included in our study. Stroke type, NIHSS, pre-existing comorbidities, and functional outcomes were collected according to a pre-defined system. A smoker was defined as active tobacco smoking, more than one cigarette per day for more than 6 months at the time of stroke—a binary variable to distinguish between smokers and non-smokers. Due to assessment of young stroke, we excluded patients aged <20 and >50 years. Those who stopped smoking more than 2 years before stroke or did not complete the 3-month follow-up were excluded. Finally, 9,087 young stroke patients were included in this cohort study.

### Main Outcome Measures and Statistical Analysis

The potential confounding factors in the present study, including age, gender, hypertension, diabetes mellitus, hyperlipidemia, heart disease, previous stroke, uremia, alcohol consumption, stroke type, and NIHSS score, were defined in accordance with the consensus TSR criteria (https://www.ahajournals.org/doi/suppl/10.1161/CIRCULATIONAHA.110.936526).

All descriptive data are expressed as numbers (*N*) and percentages (%) of patients. Hazard ratios (HRs) and 95% confidence intervals (CIs) were assessed by Cox proportional hazards models in univariate analyses to compare demographic variables and risk factor prevalence at baseline and, in stratified multivariable analyses, to detect the independent predictors of mortality. HRs were adjusted for 11 variables: age, gender, hypertension, diabetes mellitus, hyperlipidemia, heart disease, previous stroke, uremia, alcohol consumption, stroke type, and NIHSS score. Statistical significance was considered at a *p*-value of <0.05.

NIHSS and mRS scores were used to assess stroke severity and functional outcomes. An mRS score of 3 or higher was considered an unfavorable outcome. Then, we used logistic regression analysis to calculate the odds ratios (ORs) for the evaluation of potential factors associated with discharging stroke patients for poor functional outcome (mRS score of 3–5) in the derivation group. Covariates, including age, gender, hypertension, diabetes mellitus, hyperlipidemia, heart disease, previous stroke, uremia, alcohol consumption, stroke type, and NIHSS score, were adjusted. Statistical significance was considered at a *p*-value of <0.05.

### Data Availability Statement

The TSR raw data are not publicly available and are maintained by the Management Office for Health Data (Dry Lab), China Medical University Hospital (http://stroke.cmuh.org.tw/). All researchers can submit their proposals to the research committee of the TSR (taiwanstrokeregistry@gmail.com). After permission, the results will be sent to investigators.

## Results

### Baseline Characteristics of Young Stroke Patients

In the TSR, 9,087 young stroke patients were identified, including 4,303 smokers and 4,784 non-smokers. The smoker group had an older age and a higher stroke severity (NIHSS on admission) than the non-smoker group (Table 1). Compared with the non-smoker group, the smoker group had higher proportions of certain stroke risk factors, such as hypertension, diabetes, hyperlipidemia, previous stroke, and alcohol consumption.

**Table 1:** Baseline characteristics of smokers/non-smokers and stroke risk factors, *n* (%).

	**Non-smokers**	**Smokers**	***p***
	**(*n* = 4,784)**	**(*n* = 4,303)**	**(Smokers vs. non-smokers)**
**Gender**			<0.0001
Women	2,551 (53.32)	403 (9.37)	
Men	2,233 (46.68)	3,900 (90.63)	
**Age (years)**	43.82 (38.05, 47.53)	44.67 (39.84, 47.69)	<0.0001
Hypertension	2,831 (59.56)	2,791 (65.93)	<0.0001
Diabetes	1,027 (21.85)	961 (23.25)	0.0472
Hyperlipidemia	857 (17.91)	980 (22.77)	<0.0001
Heart disease	694 (14.91)	542 (13.29)	0.1102
Previous stroke	472 (10.09)	440 (10.71)	0.0296
Uremia	124 (2.70)	68 (1.70)	0.004
Alcohol	322 (6.74)	1,856 (43.75)	<0.0001
**NIHSS score upon admission**	4 (1, 11)	4 (2, 11)	0.0041
0–5	2,719 (56.84)	2,470 (57.40)	
6–10	717 (14.99)	677 (15.74)	
11–15	370 (7.74)	363 (8.44)	
16–20	252 (5.27)	223 (5.19)	
>20	624 (13.05)	567 (13.19)	
**Stroke type**			<0.0001
Infarct	2,548 (54.64)	2,503 (59.09)	
TIA	330 (7.08)	264 (6.23)	
ICH	1,354 (29.04)	1,153 (27.22)	
SAH	410 (8.79)	308 (7.27)	
Other	21 (0.45)	8 (0.19)	

*NIHSS, National Institutes of Health Stroke Scale; ICH, intracranial hemorrhage; SAH, subarachnoid hemorrhage; and TIA, transient ischemic attack*.

### Smoking and Mortality in Young Stroke Patients

No significant association was observed between smoking categories and mortality (adjusted HR, 0.89; 95%CI, 0.72–1.10) in the multivariate Cox regression model (Table 2). We further analyzed the adjusted HR values stratified by gender. The adjusted HRs in the male and female groups were 0.91 (95%CI 0.71–1.15) and 0.78 (95%CI 0.47–1.28), respectively. In smokers, higher mortality was noted only in the intracerebral hemorrhage (ICH) group (HR, 1.25; 95%CI, 1.03–1.51). However, after adjustment, the difference in ICH risk between smokers and non-smokers remains not statistically significant (adjusted HR, 1.08; 95%CI, 0.81–1.44).

**Table 2:** Mortality and hazard ratio (HR) for stroke events in smokers compared to non-smokers in all stroke patients.

	**Deaths**	**Mortality (per 1,000 person-days)**	**Crude HR**	**(95% CI)**	**Adjusted HR**	**(95% CI)**
**Overall**
Non-smoker	405	0.53	1	(Reference)	1	(Reference)
Smoker	372	0.50	0.94	0.82–1.09	0.89	0.72–1.10
**Male**
Non-smoker			1	(Reference)	1	(Reference)
Smoker	331	0.49	0.95	0.79–1.14	0.91	0.71–1.15
**Female**
Non-smoker			1	(Reference)	1	(Reference)
Smoker	41	0.61	1.10	0.79–1.54	0.78	0.47–1.28
**Stroke type**
**Infarct**
Non-smoker	109	0.25	1	(Reference)	1	(Reference)
Smoker	77	0.16	0.65	0.49–0.87^**^	0.79	0.54–1.16
**TIA**
Non-smoker	4	0.08	1	(Reference)	1	(Reference)
Smoker	1	0.02	-	-	-	-
**ICH**
Non-smoker	204	1.04	1	(Reference)	1	(Reference)
Smoker	226	1.37	1.25	1.03–1.51^*^	1.08	0.81–1.44
**SAH**
Non-smoker	79	1.36	1	(Reference)	1	(Reference)
Smoker	64	1.50	1.06	0.76–1.47	0.72	0.39–1.33
**Other**
Non-smoker	2	0.49	1	(Reference)	1	(Reference)
Smoker	0	0	-	-	-	-

* *Indicating P <0.05*;

** *P <0.001*.

*Manually adjusted for age, gender, hypertension, diabetes mellitus, hyperlipidemia, heart disease, previous stroke, uremia, alcohol consumption, stroke type and NIHSS score*.

*HR, hazard ratio; ICH, intracranial hemorrhage; NIHSS, National Institutes of Health Stroke Scale; SAH, subarachnoid hemorrhage; and TIA, transient ischemic attack*.

### Smoking and the Functional Outcome in Young Stroke Patients

The poor functional outcome (with the mRS score of 3–5) between the two cohorts was assessed (Table 3). For NIHSS scores ranging between 16 and 20, adjusted ORs were only significant in smokers (OR, 2.75, 95% CI, 1.12– 6.77, respectively).

**Table 3:** Crude and adjusted ORs with 95% CI for the 3-months outcome (mRS 3–5 vs. 0–2).

**At 3 months**	**Crude**	**Adjusted**
**Overall**	1.13 (0.98–1.18)	1.03 (0.9–1.08)
NIHSS score: 0–5	1.00 (0.71–1.41)	0.97 (0.62–.51)
NIHSS score: 6–10	0.79 (0.57–1.12)	0.95 (0.60–1.52)
NIHSS score: 11–15	1.02 (0.66–1.58)	1.57 (0.83–2.96)
NIHSS score: 16–20	1.82 (1.02–3.28)^*^	2.75 (1.12–6.77)^*^
NIHSS score: > 20	1.60 (0.93–2.75)	1.73 (0.78–3.83)

* *Indicates a significant (p <0.05) for the long-term outcome (mRS 3–5 vs. 0–2) in smokers compared with non-smokers stratified by the NIHSS score*.

*Manually adjusted for age, gender, hypertension, diabetes mellitus, hyperlipidemia, heart disease, previous stroke, uremia, alcohol consumption, stroke type and NIHSS score*.

*NIHSS, National Institutes of Health Stroke Scale*.

## Discussion

In our community-based longitudinal study in young stroke, a total of 9,087 strokes were identified among patients aged ≤ 50 years. We observed a gender difference between smokers and non-smokers. Smokers were associated with the higher severity of the initial stroke (NIHSS score upon admission) and more ischemic stroke. In addition, modifiable risk factors are prevalent in smokers, including hypertension (66.41%), diabetes (23.59%), hypercholesterolemia (31.52%), previous stroke (11.50%), and alcohol consumption (42.35%). According to the stratified analysis, smoking has detrimental effects on post-stroke outcomes. However, only lower crude ratios for mortality, not adjusted HRs, were observed in the smoker group. Smoking cessation is recommended to improve post-stroke functional outcome.

Approximately 10% of strokes occur at ages ≤ 50 years, and the proportion of strokes in young adults has increased over time (3, 4). Despite their more favorable stroke outcomes, younger adult patients still have an obvious socioeconomic consequence because a large proportion of them are at a higher risk of future cardiovascular events and labor productivity loss. Besides this, the burden of disease is heavier in the case of recurrent events. Stroke is far more common in the geriatric population, and associations between risk factors for acute stroke and the clinical outcomes are stronger among older adults (1–4). However, the knowledge gleaned from research on older adults cannot always be applied to younger adults. Lutski et al. show that young adults have a high prevalence of modifiable vascular risk factors and, in particular, a high rate of smoking (17). In our study, we found that most strokes in young smokers were related to the existence of traditional stroke risk factors. There are several reasons why smokers may have a higher risk of developing stroke. Smoking increases the risk of complex atherosclerotic cardiovascular events, including deleterious effects on the endothelial function, inflammation, lipids, and thrombosis (9, 10). Nicotine exposure could also induce a reduction in insulin release, and negatively affect insulin action, suggesting that nicotine could be a cause for the development of insulin resistance. Therefore, young stroke patients who smoked are more likely to have those comorbidities.

Initially, the term “smoker's paradox” was an observational phenomenon of an unexpected favorable outcome in smokers who experienced acute myocardial infarction and had a smoking duration of over 25 years (9). Recent studies in stroke indicate a strong positive correlation between recanalization and smokers, indicating that thrombolytic therapy acts more effectively in smokers (11, 12). However, they had some shortcomings, including short observation durations or their patients' low clinical risk profiles and low NIHSS scores on admission. In addition, those studies ignore that smoking is associated with a younger age of stroke. The risk of recurrent stroke after the first stroke is about 10% at 1 year, 25% at 5 years, and 40% at 10 years, and about 40% of stroke survivors become disabled after stroke (18). Recurrent strokes often have a higher rate of death and disability. In our present study, we only investigated young stroke patients, and the data suggest that smoking is associated with higher severity of the initial stroke, more modifiable risk factors, and poor functional outcome recorded at 3 months after stroke. Therefore, the smoker's paradox is misleading.

The difference in the incidence of stroke between the two sexes are well-established. Several epidemiological studies report higher age-specific stroke rates in men (19–21). However, women still experience more frequent stroke events because of their increased longevity and high stroke incidence at older ages. The higher prevalence of traditional vascular risk factors, including hypertension, diabetes mellitus, and hyperlipidemia in middle-aged men may contribute to this result (22, 23). Our findings are consistent with those of a meta-analysis and certain community-based reports on the relationship between stroke and sex. More interestingly, age-specific stroke rates are higher in men, and the higher prevalence of traditional vascular risk factors is associated with smoking.

Patients who cease smoking are expected to live longer and be less likely to develop tobacco-related diseases, including coronary heart disease, stroke, cancer, and pulmonary disease (24–27). In 1997, Taiwan established its first comprehensive policy package for tobacco control, the THPA, to include pictorial health warnings, smoke-free worksites and restaurants, and a ban on most tobacco advertising (24, 25). In 2018, Taiwan recorded an adult smoking rate of 13%—its lowest rate since 1990. This resulted from the efforts of the government to discourage smoking since the enactment of the THPA in 1997. Taiwan was the second Asian country after Bhutan to institute an indoor smoking ban. However, Taiwan's tobacco price still remains too low, and now, the Taiwan government only levies one package of 20 cigarettes at NT$10 (US$0.3). According to a World Bank study, a 10% rise in the cigarette price leads to a 4–8% reduction in cigarette consumption. Therefore, it is imperative to raise the price of tobacco.

The TSR provides the opportunity to investigate these issues owing to its large sample size, the homogeneous demographic characteristics and clinical phenotypes of the study subjects, and the standard diagnostic workup (16, 28, 29).

However, the present study has a number of limitations that need to be addressed. First, the baseline characteristics of the study groups were only obtained in the hospitals; hence, certain unadjusted potential confounders may still exist. Second, the sample sizes were sufficiently large, representing 3% of the total adult population in Taiwan. However, our cohort consisted of participants with an above-average socioeconomic status engaging in a medical screening program. This may affect the generalizability of our findings. Third, the smoking paradox refers to a better outcome following intravenous thrombolysis as well as endovascular therapy in smokers. However, TSR does not include data on patients who accepted endovascular therapy, leading to the possible underestimation of the reperfusion rate. Fourth, our body begins a series of positive changes that continue for years after quitting smoking. Being smoke-free not only adds years to your life, but also greatly improves your chances of a disease-free old age. Our data included patients who stopped smoking more than 2 years before stroke defined as past smokers, leading to the possible underestimation of the benefit of smoking cessation.

## Conclusion

In conclusion, this study uncovers an intriguing relation between smoking and young stroke. High smoking rates are found among male young stroke patients, compared with much lower rates among females. Smoking is associated with higher comorbidities and poor long-term function outcomes. Smoking cessation would substantially reduce those effects. This implies that physicians should put more efforts into reducing cigarette smoking in Taiwan.

## Data Availability Statement

Publicly available datasets were analyzed in this study. This data can be found here: The TSR raw data in this study are managed by the Management Office for Health Data (Dry Lab), China Medical University Hospital (http://stroke.cmuh.org.tw/). All researchers can submit their proposals to the research committee of the TSR (taiwanstrokeregistry@gmail.com). After study proposals are accepted, the results will be sent back to investigators.

## Ethics Statement

The studies involving human participants were reviewed and approved by the China Medical University Hospital Institutional Review Board. Written informed consent for participation was not required for this study in accordance with the national legislation and the institutional requirements.

## Author Contributions

P-LC, W-LC, P-YY, C-YW, M-JT, YS, C-HL, J-TL, T-CL, L-ML, M-CL, C-LLin, and H-KW: designed research. C-LLia, H-JC, Y-CL, C-CW, C-HT, and H-KW: analyzed data. H-KW and CH wrote the paper. All authors contributed to the article and approved the submitted version.

## Conflict of Interest

The authors declare that the research was conducted in the absence of any commercial or financial relationships that could be construed as a potential conflict of interest.

## Publisher's Note

All claims expressed in this article are solely those of the authors and do not necessarily represent those of their affiliated organizations, or those of the publisher, the editors and the reviewers. Any product that may be evaluated in this article, or claim that may be made by its manufacturer, is not guaranteed or endorsed by the publisher.
