# Supplementary material for: Smoking Status and Functional Outcomes in Young Stroke
Source: Front Neurol. 2021 Sep 1;12:658582. doi: 10.3389/fneur.2021.658582 (PMC8440842; doi:10.3389/fneur.2021.658582)
Supplement: Supplementary file 1 [file Data_Sheet_1.docx]

**Appendix I: Co-Authors**

**List of TSR Investigators:**

**China Medical University Hospital:** Chung-Hsiang Liu (Principal Investigator),

Chon-Haw Tsai, Wei-Shih Huang, Chung-Ta Lu, Tzung-Chang Tsai, Chun-Hung

Tseng, Kang-Hsu Lin, Woei-Cherng Shyn, Yu-Wan Yang, Yen-Liang Liu,

Der-Yang Cho, Chun-Chung Chen

**National Taiwan University Hospital:** Jiann-Shing Jeng (Principal Investigator), Sung-Chun Tang, Li-Kai Tsai, Shin-Joe Yeh

**E-Da Hospital / I-Shou University:** Shih-Pin Hsu (Principal Investigator), Han-Jung Chen, Cheng-Sen Chang, Hung-Chang Kuo, Lian-Hui Lee, Huan-Wen Tsui, Jung-Chi Tsou, Yan-Tang Wang, Yi-Cheng Tai,Kun-Chang Tsai, Yen-Wen Chen, Kang Lu, Po-Chao Liliang, Yu-Tun Tsai, Cheng-Loong Liang, Kuo-Wei Wang, Hao-Kuang Wang, Jui-Sheng Chen,  Po-Yuan Chen, Cien-Leong Chye, Wei-Jie Tzeng, Pei-Hua Wu

**National Cheng Kung University Hospital:**  Chih-Hung Chen (Principal Investigator), Pi-Shan Sung, Han-Chieh Hsieh, Hui-Chen Su

Shin Kong WHS Memorial Hospital: Hou-Chang Chiu (Principal Investigator), Li-Ming Lien, Wei-Hung Chen, Chyi-Huey Bai, Tzu-Hsuan Huang, Chi-Ieong Lau,Ya-Ying Wu, Hsu-Ling Yeh, Anna Chang

**Kaohsiung Veterans General Hospital:** Ching-Huang Lin (Principal Investigator), Cheng-Chang Yen

**Kaohsiung Medical University Chung-Ho Memorial Hospital:** Ruey-Tay Lin

(Principal Investigator), Chun-Hung Chen, Gim-Thean Khor, A-Ching Chao,

Hsiu-Fen Lin, Poyin Huang

**Chi Mei Medical Center:** Huey-Juan Lin (Principal Investigator), Der-Shin Ke,

Chia-Yu Chang, Poh-Shiow Yeh, Kao-Chang Lin, Tain-Junn Cheng, Chih-Ho Chou, Chun-Ming Yang, Hsiu-Chu Shen

**Chung Shan Medical University Hospital:** An-Chih Chen (Principal Investigator), Shih-Jei Tsai, Tsong-Ming Lu, Sheng-Ling Kung, Mei-Ju Lee, Hsi-Hsien Chou

**Show Chwan Memorial Hospital:** Hsin-Yi Chi (Principal Investigator), Chou-Hsiung Pan, Po-Chi Chan, Min-Hsien Hsu, Wei-Lun Chang,Ya-Ying Wu , Zhi-Zang Huang , Hai-Ming Shoung,Yi-Chen Lo, Fu-Hwa Wang

**Cheng Hsin General Hospital:** Ta-Chang Lai (Principal Investigator), Jiu-Haw Yin,

Chung-JenWang, Kai-ChenWang, Li-Mei Chen, Jong-Chyou Denq

**En Chu Kong Hospital:** Yu Sun (Principal Investigator), Chien-Jung Lu, Cheng-Huai Lin, Chieh-Cheng Huang, Chang-Hsiu Liu, Hoi-Fong Chan

**Far Eastern Memorial Hospital:** Siu-Pak Lee (Principal Investigator)

**Kuang Tien General Hospital:** Ming-Hui Sun (Principal Investigator),

Li-Ying Ke

**Taichung Veterans General Hospital:** Po-Lin Chen (Principal Investigator),

Yu-Shan Lee

**Ditmanson Medical Foundation Chia-Yi Christian Hospital:** Sheng-Feng Sung(Principal Investigator), Cheung-Ter Ong, Chi-Shun Wu, Yung-Chu Hsu, Yu-Hsiang Su, Ling-Chien Hung

**Tri-Service General Hospital:** Jiunn-Tay Lee (Principal Investigator), Jiann-Chyun Lin, Yaw-Don Hsu, Jong-Chyou Denq, Giia-Sheun Peng, Chang-Hung Hsu, Chun-Chieh Lin, Che-Hung Yen, Chun-An Cheng, Yueh-Feng Sung, Yuan-Liang Chen, Ming-Tung Lien, Chung-Hsing Chou, Chia-Chen Liu, Fu-Chi Yang, Yi-ChungWu, An-Chen Tso, Yu- Hua Lai, Chun-I Chiang, Chia-Kuang Tsai, Meng-Ta Liu, Ying-Che Lin, Yu-Chuan Hsu

**Cathay General Hospital:** Tsuey-Ru Chiang (Principal Investigator),

Mei-Ching Lee, Pai-Hao Huang, Sian-King Lie, Pin-Wen Liao, Jen-Tse Chen

**Changhua Christian Hospital:** Mu-Chien Sun (Principal Investigator), Tien-Pao Lai, Wei-Liang Chen, Yen-Chun Chen, Ta-Cheng Chen, Wen-Fu Wang, Kwo-Whei Lee, Chen-Shu Chang, Chien-Hsu Lai, Siao-Ya Shih, Chieh-Sen Chuang, Yen-Yu Chen, Chien-Min Chen

**Taipei Tzuchi Hospital, Buddhist Tzuchi Medical Foundation:** Shinn-Kuang Lin (Principal Investigator, School of Medicine, Tzuchi University, Hualien, Taiwan), Yu-Chin Su, Cheng-Lun Hsiao, Fu-Yi Yang, Chih-Yang Liu, Han-Lin Chiang, Ser-Chen Fu

**Min Sheng General Hospital:** Chun-Yuan Chang (Principal Investigator), I-sheng Lin,Chung-Hsien Chien,Yang-Chuang Chang

**Lin Shin Hospital:** Ping-Kun Chen (Principal Investigator), Pai-Yi Chiu

**National Taiwan University Hospital Yunlin Branch:** Yu-Jen Hsiao (Principal Investigator), Chen-Wen Fang

**Landseed Hospital:** Yu-Wei Chen (Principal Investigator), Kuo-Ying Lee, Yun-Yu Lin, Chen-Hua Li, Hui-Fen Tsai, Chuan-Fa Hsieh, Chih-Dong Yang, Shiumn-Jen Liaw, How-Chin Liao

**Cheng Ching General Hospital:** Shoou-Jeng Yeh (Principal Investigator), Ling-Li

Wu, Liang-Po Hsieh, Yong-Hui Lee, Chung-Wen Chen

**China Medical University Beigang Hospital:** Chih-Shan Hsu(Principal Investigator),Ye-Jian-Jhih, Hao-Yu Zhuang, Yan-Hong Pan, Shin-An Shih

**Taipei Medical University -Wan Fang Hospital:** Chin-I Chen (Principal Investigator), Jia-Ying Sung, , Hsing-Yu Weng, Hao-Wen Teng, Jing-Er Lee, Chih-Shan Huang, Shu-Ping Chao

**Taipei Medical University Hospital:** Rey-Yue Yuan (Principal Investigator),

, Jau- Jiuan Sheu, Jia-Ming Yu, Chun-Sum Ho, Ting-Chun Lin

**Kuang Tien General Hospital Dajia Division:** Shih-Chieh Yu(Principal Investigator)

**Changhua Christian Hospital Yunlin Branch:** Jiunn-Rong Chen (Principal

Investigator), Song-Yen Tsai

**Chang Bing Show Chwan Memorial Hospital:** Cheng-Yu Wei (Principal Investigator), Tzu-Hsuan Huang, Chao-Nan Yang, Chao-Hsien Hung, Ian Shih

**Lotung Poh Ai Hospital:** Hung-Pin Tseng (Principal Investigator), Chin-Hsiung Liu, Chun-Liang Lin, Hung-Chih Lin, Pi-Tzu Chen

**Taipei Medical University - Shuang Ho Hospital:** Chaur-Jong Hu (Principal Investigator), Nai-Fang Chi, Lung Chan

**Taipei Veterans General Hospital & National Yang-Ming University School of Medicine:**  Chang-Ming Chern (Principal Investigator),   Chun-Jen Lin,  Shuu-Jiun Wang, Li-Chi Hsu,  Wen-Jang Wong, I-Hui Lee, Der-Jen Yen, Ching-Piao Tsai, Shang-Yeong Kwan, Bing-Wen Soong, Shih-Pin Chen, Kwong-Kum Liao, Kung-Ping Lin, Chien Chen, Din-E Shan, Jong-Ling Fuh, Pei-Ning Wang, Yi-Chung Lee, Yu-Hsiang Yu, Hui-Chi  Huang,  Jui-Yao Tsai

**Chi Mei Medical Center, Liouying:** Ming-Hsiu Wu (Principal Investigator),

Shi-Cheng Chen, Szu-Yi Chiang, Chiung-Yao Wang

**Buddhist Dalin Tzu Chi General Hospital:** Ming-Chin Hsu (Principal Investigator)

**St. MARTIN DE PORRES HOSPITAL:** Chien-Chung Chen (Principal Investigator), Po-Yen Yeh, Yu-Tai Tsai, Ko-Yi Wang

**Sin-Lau Hospital, Tainan, the Presbyterian Church in Taiwan:** Tsang-Shan Chen(Principal Investigator)

**Cardinal Tien Hospital:** Ping-Keung Yip (Principal Investigator), Vinchi Wang,

Kaw-ChenWang, Chung-Fen Tsai, Chao-Ching Chen, Chih-Hao Chen, Yi-Chien

Liu, Shao-Yuan Chen, Zi-Hao Zhao, Zhi-Peng Wei

**Yumin Medical Corporation Yumin Hospital:** Shey-Lin Wu(Principal Investigator)

**Kaohsiung Municipal Hsiao-kang Hospital:** Ching-Kuan Liu(Principal Investigator)

**Wei Gong Memorial Hospital:** Ryh-Huei Lin (Principal Investigator), Ching-Hua Chu

**Taipei City Hospital Ren Ai Branch:** Sui-Hing Yan (Principal Investigator),

Yi-Chun Lin, Pei-Yun Chen, Sheng-Huang Hsiao

**National Taiwan University Hospital Hsin-Chu Branch:** Bak-Sau Yip (Principal Investigator), Pei-Chun Tsai,Ping-Chen Chou, Tsam-Ming Kuo, Yi-Chen Lee, Yi-Pin Chiu, Kun-Chang Tsai

**Taichung Hospital Department of Health :** Yi-Sheng Liao (Principal Investigator)

**Tainan Municipal An-Nan Hospital-China Medical University:** Ming-Jun Tsai (Principal Investigator), Hsin-Yi Kao
